# Supplementary material for: Solvent-free bottom-up patterning of zeolitic imidazolate frameworks
Source: Nat Commun. 2022 Jan 20;13:420. doi: 10.1038/s41467-022-28050-z (PMC8776825; doi:10.1038/s41467-022-28050-z)
Supplement: Supplementary file 1 — Supplementary Information [file 41467_2022_28050_MOESM1_ESM.pdf]

Supplementary Information for

Solvent-Free Bottom-up Patterning of Zeolitic Imidazolate Frameworks

Yurun Miao[1,7], Dennis T. Lee[1,7], Matheus Dorneles de Mello[2,3], Mueed Ahmad[2,5], Mohammed K. Abdel-Rahman[4], Patrick Eckert,[4] J. Anibal Boscoboinik[2,5], D. Howard Fairbrother[4], Michael Tsapatsis[1,6]*

[1]Department of Chemical and Biomolecular Engineering & Institute for NanoBioTechnology, Johns Hopkins University, Baltimore, MD, USA. [2]Center for Functional Nanomaterials, Brookhaven National Laboratory, Upton, NY, USA. [3]Catalysis Center for Energy Innovation, University of Delaware, Newark, DE, USA. [4]Department of Chemistry, Johns Hopkins University, Baltimore, MD, USA. 5Department of Materials Science and Chemical Engineering, Stony Brook University, Stony Brook, NY, USA. [6]Applied Physics Laboratory, Johns Hopkins University, Laurel, MD, USA. [7]These authors contributed equally to this work. Email: tsapatsis@jhu.edu.

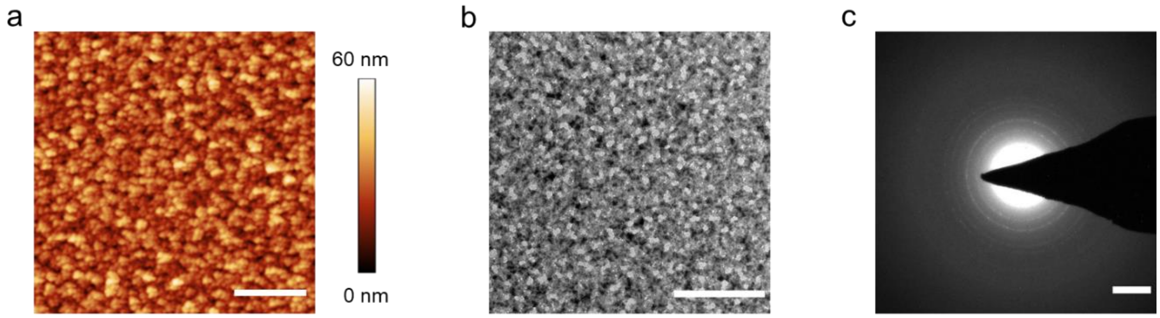

Supplementary Figure 1. (a) AFM, (b) TEM images, and (c) SAED pattern of ZIF-8 deposited in a non-irradiated area on a 50-nm-thick silicon nitride substrate. Scale bars are 1 μm in a and b, and 1 nm^{-1} in c.

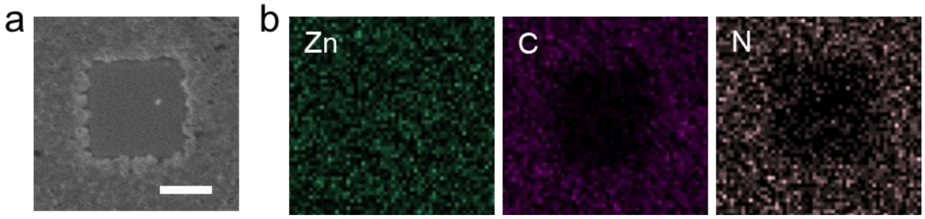

Supplementary Figure 2. (a) SEM and (b) corresponding EDS element mapping of a ZIF-8-free area in a ZIF-8 film. The ZnO wafer is sensitized by 2mIm at 50 °C for 1 h, followed by e-beam irradiation at 2 kV, 20 mC/cm^2 and then subjected to 2mIm vapor treatment at 120 °C for 15 min. Scale bar is 1 μm.

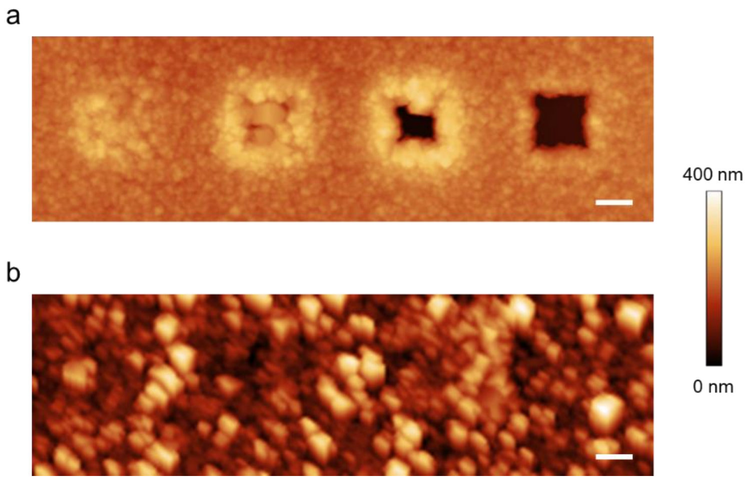

Supplementary Figure 3. AFM images of a sample after 2mIm vapor treatment (a) at 100 °C for 1 h and (b) at 120 °C for 2 h, respectively. Before the 2mIm vapor treatment, the ZnO film is sensitized at 50 °C for 1 h with 2mIm and e-beam irradiated with an electron dose of 0.5, 5, 10 and 20 mC/cm^2 in each of the four squares (from left to right). Scale bars are 1 µm.

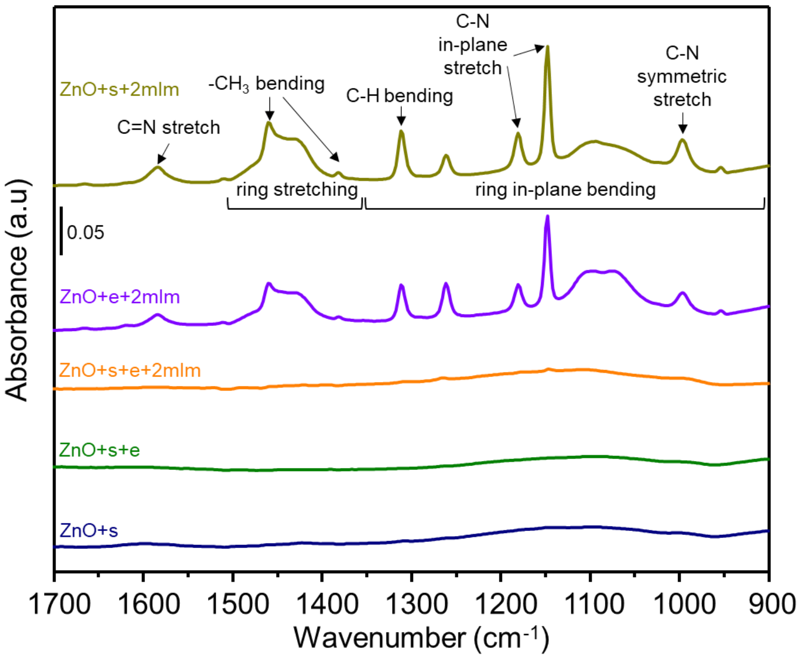

Supplementary Figure 4. IRRAS of a ZnO wafer after sequential treatment of sensitization, e-beam irradiation, and 2mIm treatment. Samples are denoted by the experimental steps: s, sensitization with 2mIm at 50 °C; e, e-beam irradiation at 2 kV, 20 mC/cm^2; 2mIm, vapor treatment with 2mIm at 120 °C for 1 h. Peaks characteristic of ZIF-8 are labelled for ZnO+s+2mIm sample.[1–3]

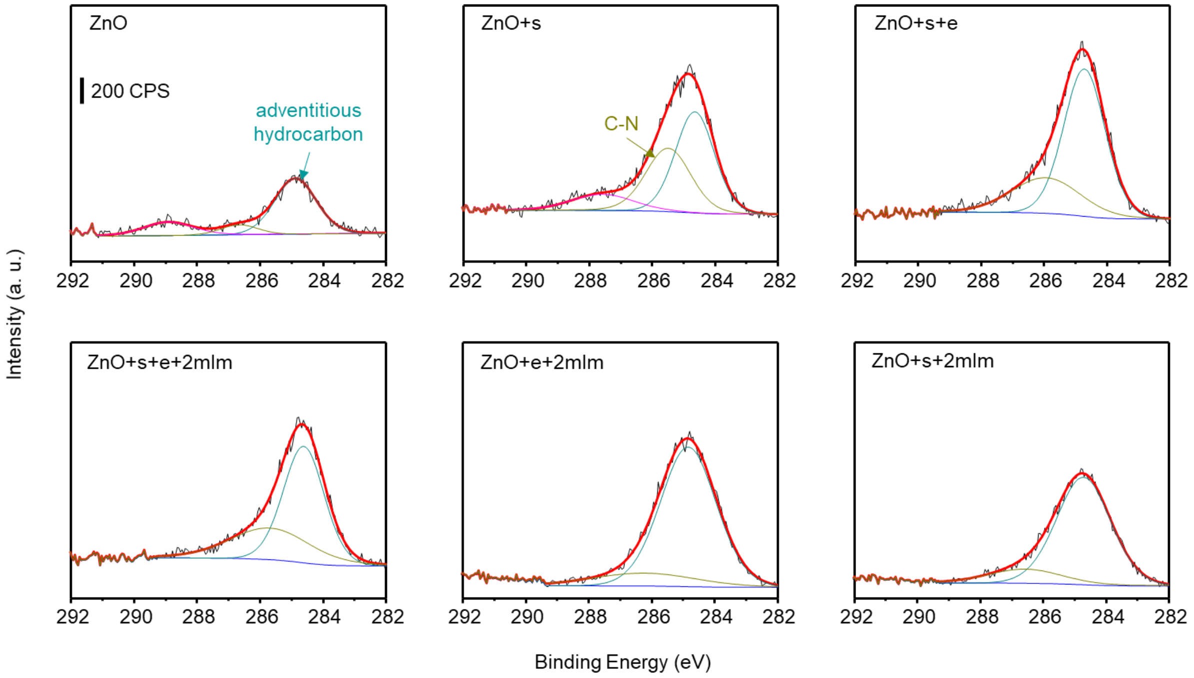

Supplementary Figure 5. C 1*s* XPS of ZnO samples after each step of treatment. Samples are denoted to indicate the processing steps used, s: sensitization with 2mIm at 50 °C for 1 h; e: e-beam irradiation at 2 kV, 20 mC/cm^2; 2mIm: vapor treatment with 2mIm at 120 °C for 15 min. In ZnO, the peak at 284.8 eV is attributed to adventitious hydrocarbon and the peaks at 286.7 and 288.9 eV are attributed to adsorbed oxygen-containing species.[4] After sensitization (ZnO+s), the peak emerging at 285.5 eV is assigned to C-N in adsorbed 2mIm linker.[5] The diminished shoulder above 288 eV in e-beam treated sample (ZnO+s+e) may suggest reduced adsorption of oxygen-containing species after e-beam irradiation due to modified surface properties.

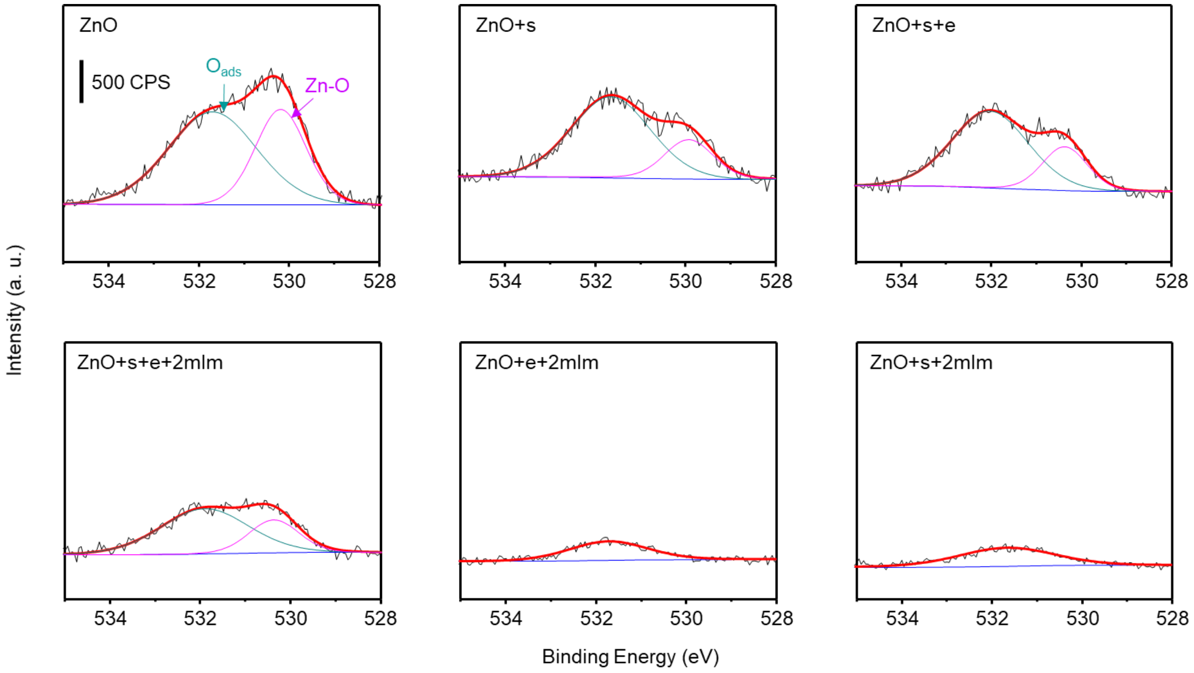

Supplementary Figure 6. O 1*s* XPS of ZnO samples after each step of treatment. Samples are denoted to indicate the processing steps used, s: sensitization with 2mIm at 50 °C for 1 h; e: e-beam irradiation at 2 kV, 20 mC/cm^2; 2mIm: vapor treatment with 2mIm at 120 °C for 15 min. The peaks at 530.0 and 531.8 eV are attributed to O^{2-} in ZnO and surface adsorbed water, respectively.[6–8] The change in the relative intensity of the two peaks after sensitization (ZnO+s) compared to ALD ZnO is likely due to adsorbed 2mIm layer covering the surface of ZnO.

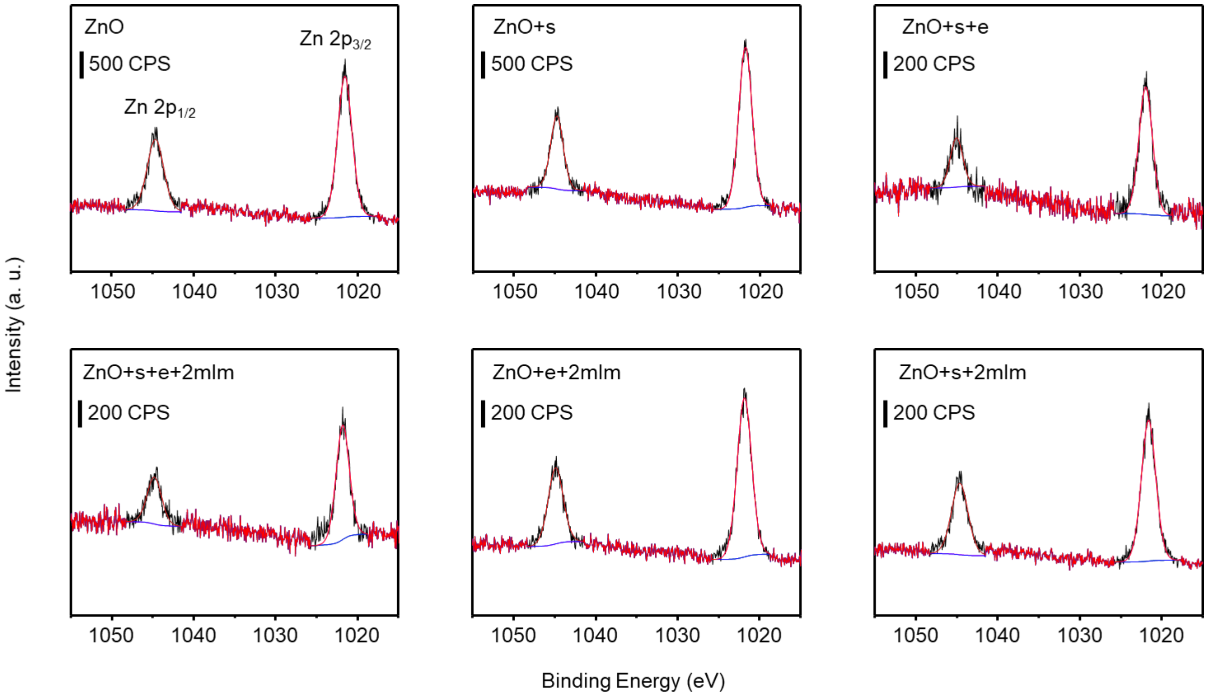

Supplementary Figure 7. Zn 2*p* XPS of ZnO samples after each step of treatment. Samples are denoted to indicate the processing steps used, s: sensitization with 2mIm at 50 °C for 1 h; e: e-beam irradiation at 2 kV, 20 mC/cm^2; 2mIm: vapor treatment with 2mIm at 120 °C for 15 min. The peaks at 1044.7 and 1021.6 eV are attributed to Zn 2$p_{1/2}$ and Zn 2$p_{3/2}$, respectively.[9]

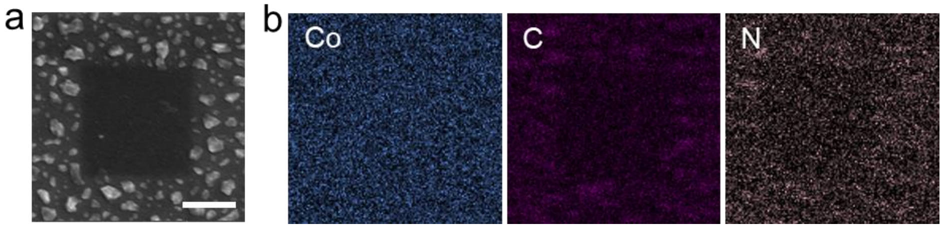

Supplementary Figure 8. (a) SEM and (b) corresponding EDS element mapping of a ZIF-67-free area in a ZIF-67 film. The CoO_x wafer is sensitized by 2mIm at 50 °C for 2 h, followed by e-beam irradiation at 2 kV, 20 mC/cm^2 and then subjected to 2mIm vapor treatment at 120 °C for 2 h. Scale bar is 1 µm.

Supplementary References

1. Low, Z. X. *et al.* Crystal transformation in zeolitic-imidazolate framework. *Cryst. Growth Des.* **14**, 6589–6598 (2014).

2. Tian, F. *et al.* Surface and stability characterization of a nanoporous ZIF-8 thin film. *J. Phys. Chem. C* **118**, 14449–14456 (2014).

3. Conrad, S. *et al.* Controlling Dissolution and Transformation of Zeolitic Imidazolate Frameworks by using Electron-Beam-Induced Amorphization. *Angew. Chemie - Int. Ed.* **57**, 13592–13597 (2018).

4. Alshammari, A. S. *et al.* Visible-light photocatalysis on C-doped ZnO derived from polymer-assisted pyrolysis. *RSC Adv.* **5**, 27690–27698 (2015).

5. Luanwuthi, S., Krittayavathananon, A., Srimuk, P. & Sawangphruk, M. In situ synthesis of permselective zeolitic imidazolate framework-8/graphene oxide composites: Rotating disk electrode and Langmuir adsorption isotherm. *RSC Adv.* **5**, 46617–46623 (2015).

6. Kotsis, K. & Staemmler, V. Ab initio calculations of the O1s XPS spectra of ZnO and Zn oxo compounds. *Phys. Chem. Chem. Phys.* **8**, 1490–1498 (2006).

7. Wöll, C. The chemistry and physics of zinc oxide surfaces. *Prog. Surf. Sci.* **82**, 55–120 (2007).

8. Zhang, X. *et al.* Effect of aspect ratio and surface defects on the photocatalytic activity of ZnO nanorods. *Sci. Rep.* **4**, 4–11 (2014).

9. Zhang, L. *et al.* ZIF-8 derived ZnO/Zn6Al2O9/Al2O3 nanocomposite with excellent photocatalytic performance under simulated sunlight irradiation. *New J. Chem.* **43**, 2990–2999 (2019).
